# Supplementary material for: Learning curve and surgical outcome of robotic assisted colorectal surgery with ERAS program
Source: Sci Rep. 2022 Nov 29;12:20566. doi: 10.1038/s41598-022-24665-w (PMC9709162; doi:10.1038/s41598-022-24665-w)
Supplement: Supplementary file 2 — Supplementary Information 2. [file 41598_2022_24665_MOESM2_ESM.zip › RAL Raw data-Ñ[▒K/107/HS_ñw╝╨░O▒Kñσ.pdf]

正式摘要(電子簽章：00000223685835)		
W52 / W52　☐英文標題(注意:無英文姓名-請補病人基本檔!)	☐【讀取】憑證	

臺中榮民總醫院

出院病歷摘要

轉入號 :　　　　轉出號 :

[redacted]

**(8)**流水編號	
**(9)**入院日期	110/08/18 (2021) 直腸外科 W52 - 171
**(10)**轉科日期	
**(11)**出院日期	110/08/27 (2021)　住院天數計 9 日

診斷

- 入院 :1. Colon Cancer, c T3 N1 M0, Stage IIIB
  2. Benign prostate hypertropy
  3. Hypertension
  4. Old cerebral vascular artery disease under plavix for secondary prevention
- 出院 :1. Rectum Cancer 10 cm FAV, c T3 N1 M0,
  s/p robotic low anterior resection on 2021/08/20
  pT3N1a(1/9)M0, MD.AI-, PI-, TuDp-, CRM 2cm-, DisMr:1.6cm-, ExNd- stage IIIA
  2. Benign prostate hypertropy
  3. Hypertension
  4. Old cerebral vascular artery disease under plavix for secondary prevention

主訴

- Bloody stool about 2 weeks ago

病史

- This 85 y/o male patient had history of :
  1. Colon Cancer, c T3 N1 M0, Stage IIIB
  2.Benign prostate hypertropy
  3. Hypertension
  4. Old cerebral vascular artery disease under plavix for secondary prevention

  This patient had history of anal bleeding every year. He had colonoscopy on 8/12 : rectum ca 10 ca FAV. Therefore he had abdomen CT on 8/16: 1. rectum tumor, 2. stomach fundus thickening.
  This time he was admitted for surgery survey. Esophago-gastroscopy was arranged for gastric lesion.

體檢發現

-
  - General appearance :
    - easy looking , conscious clear , without cardiopulmonary distress
    - Vital signs自動帶入date 體溫(BT) 脈搏(P) 呼吸(R) 血壓(BP) 身高(BH) 體重(BW) BMI
      20210818 36.3℃ 83次/分 19次/分 133/63mmHg 168cm 68kg 24.1
    - Vital signs: B.P. 133/63 mmHg , P.R. 83 /min , R.R. 19 /min , B.T. 36.3 ℃
  - HEENT :
    - Eyes: no anemic conjunctiva, no icteric sclera, normal EOM
    - ENT: no otorrhea, no rhinorrhea, no erythematous mucosa, no injected tonsil, no deformity
  - Neck: supple, freely movable, trachea midline,no deformity, no engorged jugular vein , no palpable thyroid or lymph node enlargement
  - Chest:
    - Inspection: Grossly normal, symmetric and fully expansion, no accessory muscle used, no operation scar, no spider angiomata, no gynecomastia
    - Palpation: no palpable mass or nodule, no subcutaneous emphysema, no tenderness
    - Ausculation: clear breathing sound
  - Heart:

-
    - Palpation: no thrill, no heave
    - Percussion: normal heart size
    - Ausculation: regular heart beat, normal S1, S2, no gallop, no murmur
  - Extremities: No deformity, full ranged of motion without limitation, no clubbing finger or nail cyanosis, no pitting edema, no ulcer or pigmentation, no obvious skin lesion, no varicose vein, no bone, joint or muscle tenderness

CRS CONDITION

- Karnofsky performance status: (100) Normal, no complaints, no evidence of disease
- PE of Abdomen:
- CEA: 3.77 ng/ml
- Imaging Studies:
- Colonoscope report: Colonscopy was up to S-colon. Tumor was noted at 10cm FAV, S/p biopsy.
- Sono , CT of abdomen and chest Films:Impression:
  1. R/I S-colon cancer. Please correlate with colonoscopy.
  2. Other findings, as above description
- Figures:
  1. colon & rectum

手術日期及方法

- 手術日期及方法20210820 Robot Assisted Colectomy(L)

住院治療經過

- After surgery, he recovered well, and take diet smoothly. Foley was removed after medication. He was discharged OPD f/u.

合併症

一般檢查

- 累積報告 :

DATE	TIME	ALB	TP	BIL.T	BIL.D	ALKP	AST	ALT	LDH	AMY	LIPASE	NH3	Ferritin	UA
1100812	1103						16	11						
1100818	1446	3.6		0.4	0.1	52	13	11	143					
1100820	1412			0.87				8						
1100823	0955	3.0		0.42				4						

DATE	TIME	WBC	RBC	HGB	HCT	MCV	PLT	BLST	NEUT	BAND	SEG	LYM	MON	EOS	BAS
1100812	1103	9990	3.96	13.2	38.3	96.7	187		90.8			5.1	3.7	0.2	0.2
1100818	1446	9710	3.67	12.1	35.8	97.5	210		83.8			7.4	7.3	1.3	0.2
1100820	1413	14930		10.7	31.1		170		90.8			3.6	5.4	0.1	0.1
1100823	0959	9620		10.1	29.3		172		86.1			5.2	5.8	2.7	0.2

DATE	TIME	NA	K	CL	BUN	CREAT	eGFR	CA	Mg	P	CRP	ESR	PCT	D-Dimer	LACTATE
1100812	1103	138	4.2	102	20	1.59		10.1							
1100818	1446	139	4.1		22	1.21		10.0							
1100820	1412	137	4.2	107	15	1.25	58.35	8.7							
1100823	0955	141	3.4		16	1.14	64.89	8.5							

DATE	TIME	GLUCOSE	HbA1C	CHOL	TG	HDL	LDL	KETONE	CK	CKMB	TRO-T	PT-P	APTT-P	NT-pro BNP
1100812	1103		6.2				67					10.7	27.2	
1100818	1446	104										10.7	27.5	
1100823	0948											11.5	38.2	

DATE	TIME	SP-GR	PH	PROT	GLUC	KETON	BILI	UROB	OB	NITR	LEU	RBC	WBC	EPCEL
1100822	1204	1.017	7.0	1+	>1000.0	Trace	Negative	0.2	3+	Negative	2+	3+	10-20	0-1

特殊檢查

病理報告

- 活體組織切片病理報告 手術日期：**110/08/20** 病理號：**S1025900**

診斷醫師:王任卿 報告日期:110/08/25
Pathologic diagnosis:
1. Rectum, robot assisted colectomy --- Adenocarcinoma, moderately differentiated, arising in a tubulovillous adenoma. Tumor invaded to perirectal soft tissue, and metastasized to perirectal lymph node

- (1/9). The bilateral cut ends are free of tumor.
2. IMA root, dissection --- Unremarkable fibroadipose tissue.
3. Rectum, cut end, distal, resection --- Unremarkable. No tumor seen.
Ancillary study for diagnosis:
IHC stains for PMS2 and MSH6 are done on section E. Both above markers are preserved.
Prognostic and predictive factor:
1. Tumor size: 5.4x4.5 cm.
2. Depth of invasion: Tumor invades to pericolorectal tissue.
3. Histologic grade: G2: Moderately differentiated.
4. Angiolymphatic invasion: Not identified.
5. Perineural invasion: Not identified.
6. Tumor deposits (discontinuous extramural extension): Not identified.
7. Macroscopic tumor perforation: Not identified.
8. Surgical Margins:
a) Proximal Margin: Uninvolved by invasive carcinoma, distance of tumor from margin: 7 cm.
b) Distal Margin: Uninvolved by invasive carcinoma, distance of tumor from margin: 1.6 cm.
c) Radial or Mesenteric Margin: Uninvolved by invasive carcinoma, distance of tumor from margin: 2 cm.
9. Lymph node status: Involved (1/9).
10. Extranodal involvement: Not identified.
11. Tumor regression grading S/P CCRT: No known presurgical therapy.
12. Type of polyp in which invasive carcinoma arose: Tubulovillous adenoma.
13. Additional pathologic findings: Not identified.
14. Pathological TNM Stage: pT3N1a(According to the eighth edition, American Joint Committee on Cancer Staging Guidelines for Tumors).
15. TNM descriptors: Not applicable.
Gross description:
The specimen consists of 1) a segment of colon, 12.5 cm in length and 3.5 cm in proximal cut end circumference, 7 cm in distal circumference, and perirectal soft tissue. A gray tan firm annular elevated and centrally ulcerative tumor, 5.4x4.5 cm, is noted, 1.6 cm to the nearest cut end. On cross section, the tumor invades to the mesocolic soft tissue with 20 mm to the radial margin, and serosal retraction is also noted. The both cut ends are free of tumor grossly. 2) IMA root, a piece of tan yellow soft tissue, 4.4x2.7x1 cm. 3) distal cut end, a piece of tan mucosa-covered soft tissue, 1.6x1.6x0.8 cm. Representative part for section: A) proximal cut end B) distal cut end C-E) tumor F) colon G-H) specimen 2-3 J-K) lymph nodes.
Note:
1. The interpretation of MMR panel and recommended studies are as below: (1) No loss of nuclear expression of MMR proteins: low probability of microsatellite instability-high (MSI-H).
(2) Loss of nuclear expression of MLH1 and PMS2: testing for methylation of the MLH1 promoter and/or mutation of BRAF is indicated (the presence of a BRAF V600E mutation and/or MLH1 methylation suggests that the tumor is sporadic and germline evaluation is probably not indicated; absence of both MLH1 methylation and of BRAF V600E mutation suggests the possibility of Lynch syndrome, and sequencing and/or large deletion/duplication testing of germline MLH1 may be indicated).
(3) Loss of nuclear expression of MSH2 and MSH6: high probability of Lynch syndrome (sequencing and/or large deletion/duplication testing of germline MSH2 may be indicated, and, if negative, sequencing and/or large deletion/duplication testing of germline MSH6 may be indicated).
(4) Loss of nuclear expression of MSH6 only: high probability of Lynch syndrome (sequencing and/or large deletion/duplication testing of germline MSH6 may be indicated).
(5) Loss of nuclear expression of PMS2 only: high probability of Lynch syndrome (sequencing and/or large deletion/duplication testing of germline PMS2 may be indicated).
(6) Please note: There are exceptions to the above IHC interpretations. These results should not be considered in isolation, and clinical correlation with genetic counseling is recommended to

- assess the need for germline testing.
  2. This case has been peer reviewed by two doctors.
  #T-59600_2 #M-82633_2 2 1100 S0000F
  #T-C4560_2 #M-81403_2 2 1100 000000

其它

出院時情況

- 3.改門診治療

出院指示

藥名	劑量	劑量單位	用法	頻次	數量	數量單位	現況	天數	單位
(□)Takepron OD tab 30mg	1	TAB	PO	QDAC	10	TAB	TH	10	天
BethanechoL tab 25mg-JS	1	TAB	PO	TIDAC	30	TAB	TH	10	天
Ultracet tab	1	TAB	PO	QIDPRN	40	TAB	TH	10	天
Econ tab 40mg	1	TAB	PO	QID	40	TAB	TH	10	天
Through tab	1	TAB	PO	QN	10	TAB	TH	10	天

出院計劃

主治醫師 2222H 林俊余 / 住院醫師 2340H 郭連雄

正式摘要**(電子簽章：000000223685835)**

W52 / W52

doctorRank:B
